# *SlMYB1* regulates the accumulation of lycopene, fruit shape, and resistance to *Botrytis cinerea* in tomato

**DOI:** 10.1093/hr/uhac282

**Published:** 2022-12-22

**Authors:** Ziyi Yin, Jiazong Liu, Haipeng Zhao, Xiaomeng Chu, Haoqi Liu, Xiangyu Ding, Chongchong Lu, Xinyu Wang, Xiangyu Zhao, Yang Li, Xinhua Ding

**Affiliations:** State Key Laboratory of Crop Biology, Shandong Provincial Key Laboratory for Biology of Vegetable Diseases and Insect Pests, College of plant protection, Shandong Agricultural University, Taian 271018, Shandong, China; State Key Laboratory of Crop Biology, Shandong Provincial Key Laboratory for Biology of Vegetable Diseases and Insect Pests, College of plant protection, Shandong Agricultural University, Taian 271018, Shandong, China; State Key Laboratory of Crop Biology, Shandong Provincial Key Laboratory for Biology of Vegetable Diseases and Insect Pests, College of plant protection, Shandong Agricultural University, Taian 271018, Shandong, China; State Key Laboratory of Crop Biology, Shandong Provincial Key Laboratory for Biology of Vegetable Diseases and Insect Pests, College of plant protection, Shandong Agricultural University, Taian 271018, Shandong, China; State Key Laboratory of Crop Biology, Shandong Provincial Key Laboratory for Biology of Vegetable Diseases and Insect Pests, College of plant protection, Shandong Agricultural University, Taian 271018, Shandong, China; State Key Laboratory of Crop Biology, Shandong Provincial Key Laboratory for Biology of Vegetable Diseases and Insect Pests, College of plant protection, Shandong Agricultural University, Taian 271018, Shandong, China; State Key Laboratory of Crop Biology, Shandong Provincial Key Laboratory for Biology of Vegetable Diseases and Insect Pests, College of plant protection, Shandong Agricultural University, Taian 271018, Shandong, China; State Key Laboratory of Crop Biology, Shandong Provincial Key Laboratory for Biology of Vegetable Diseases and Insect Pests, College of plant protection, Shandong Agricultural University, Taian 271018, Shandong, China; State Key Laboratory of Crop Biology, College of Academy of Life Science, Shandong Agricultural University, Taian 271018, Shandong, China; State Key Laboratory of Crop Biology, Shandong Provincial Key Laboratory for Biology of Vegetable Diseases and Insect Pests, College of plant protection, Shandong Agricultural University, Taian 271018, Shandong, China; State Key Laboratory of Crop Biology, College of Academy of Life Science, Shandong Agricultural University, Taian 271018, Shandong, China; State Key Laboratory of Crop Biology, Shandong Provincial Key Laboratory for Biology of Vegetable Diseases and Insect Pests, College of plant protection, Shandong Agricultural University, Taian 271018, Shandong, China

## Abstract

Fruit lycopene, shape, and resistance are essential traits in vegetables whose final product is fruit, and they are also closely related to and strictly regulated by multiple transcription factors. Lycopene, which cannot be synthesized by the human body and can only be ingested from the outside, was important in maintaining human health. During fruit ripening and post-harvest, tomato plants face a variety of biotic or abiotic stresses, which might inflict great damage to fruit quality due to its flat shape and pointed tip during storage and transportation. Therefore, there is an urgent need for key molecular switches to simultaneously improve fruit lycopene and resistance to biotic stress during ripening. Here, we identified the MYB transcription factor *SlMYB1* in tomato plants which could bind to the promoters of lycopene synthesis-related genes, *SlLCY1, SlPSY2*, and the pathogen-related gene *SlPR5* directly, to regulate the fruit lycopene and resistance to *Botrytis cinerea* in tomato. In addition to regulating lycopene synthesis, *SlMYB1* also regulates the content of soluble sugar, soluble protein and flavonoid in tomato. What’s more, *SlMYB1* could regulate the tomato fruit shape, making it smoother or flatter to prevent skin damage caused by vibration on fruits. RNA sequencing (RNA-seq) further showed that *SlMYB1* fruit-specific expression lines had multiple differentially expressed genes compared with those from wild-type plants, suggesting that *SlMYB1* might have multiple roles in fruit nutritional quality control and resistance to stresses, which is a rare occurrence in previous studies. In summary, our results revealed that *SlMYB1* was an essential multi-functional transcription factor that could regulate the lycopene and resistance to *Botrytis cinerea*, and change the shape of fruit in tomato plants.

## Introduction

Tomato (*Solanum lycopersicum* L.) is one of the most important vegetable plants worldwide. It contains abundant nutrients such as lycopene, potassium, flavonoid, and vitamin C [1–4]. Global climate change poses growing threats of biotic (e.g. pathogens and insects) and abiotic stresses (e.g. drought) to tomato production during plant growth and development [5–7]. In addition, tomato easily loses its firm texture, being a soft fruit, thus becoming sensitive to diseases during storage and transport [8]. Therefore, identifying the genes related to stress resistance and superior fruit quality is very important in developing high-quality and disease-resistant plant varieties through selection and breeding [9, 10]. With continuous improvements in genome sequence data quality, detailed and valuable genetic information has been provided, which greatly accelerates the cloning process and analysis of the molecular mechanism of gene actions that are responsible for disease resistance and fruit quality [11–13].

Like many fruits incorporated into the human diet, tomatoes have been domesticated for centuries to meet consumer preferences, but they originally evolved to attract seed dispersers. The good flavor and abundant nutrients would boost the consumption of tomato fruit by animals to promote seed dispersal [14, 15]. Additionally, maturation-related pathways are the basic determinants of the human diet, rich in nutrition and taste [16].

*Botrytis cinerea* is a geographically widely distributed fungal pathogen of plants with a ‘necrotrophic’ lifestyle which seriously affects tomato fruits during maturation and post-harvest storage [17]. The developmental stages of *B. cinerea* start with the invasion of the host plant once conidia are formed, allowing the fungus to establish infection. It can destroy the host cells by secreting cell-wall-degrading enzymes and some toxins. Besides tomato, this fungus can infect over 200 dicot crop hosts [18]. Recently, several genes involved in resistance to *B. cinerea* in tomatoes, including *SlSKIP* [19], *SlTCP29* [20], *SlMYC2* [21], *SlARG2* [22], *SlERF2* [23], and *SlMBF1* [8] have been reported. However, few genes have been identified that can both regulate tomato fruit quality and resistance to *B. cinerea*.

**Figure 1:**
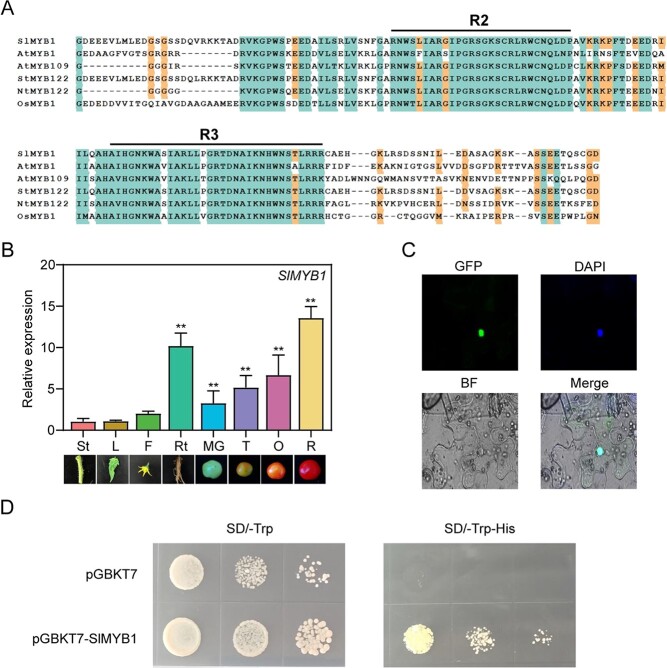
Features of the *SlMYB1* including multiple sequence alignments, tissue specific expression analysis, subcellular localization, and transcriptional activity analysis. **A** Multiple protein sequence alignments of *SlMYB1* and other R2R3-type MYB transcription factors in different plant species; other R2R3-type MYB transcription factors contain *AtMYB109*, *StMYB122*, *MtMYB122*, and *OsMYB1*. **B** The expression level of *SlMYB1* in different plant growth stages by RT-qPCR; St, stem; L, leaf; F, flower; Rt, root; MG, mature green; T, turning; O, orange; R, red. The error bars indicate stand for the SD (*n* = 3). Significant difference was determined using the Student’s *t*-test (two-samples, *P < 0.05 and **P < 0.01). **C** Subcellular localization of *SlMYB1*, which was fused to GFP to obtain the recombinant protein *SlMYB1*-GFP. **D** Transcriptional activity of *SlMYB1*; Yeast cultures were diluted and streaked onto the selective medium (−Trp-His plates), and then plates were incubated at 30°C for 3 days before the examination.

MYB transcription factors exist widely in organisms such as animals, plants, and fungi. As a large gene family, MYB transcription factors play diverse and important roles in the whole plant growth period, such as growth, development, resistance to adversity and metabolism [24, 25]. The MYB transcription factor family can be divided into four subfamilies: 4R-MYB, 3R-MYB, R2R3-MYBs, and 1R-MYB (MYB-related) [26]. R2R3-MYB class was the largest, which are involved in various biochemical pathways of primary and secondary metabolism that play roles in plant growth, development and protect them from biotic and abiotic stress. For example, *SlMYB72* regulates the content of carotenoids, chlorophylls, and also flavonoids in tomato fruits [27]. Meanwhile, *SlMYB102* can improve salt tolerance in tomatoes [28], *OsMYB1* regulates phosphate homeostasis in a steady-state condition and root development [29], whereas *StMYB122* can be induced by Benzisothiadiazole (BTH) and may regulate plant responses to the pathogen [30]. However, only some MYB proteins in tomato plants have been functionally studied, whereas a large number of them remain unidentified and uncharacterized and, therefore, were not conducive to the mining of high-quality genetic resources of tomato.

**Figure 2:**
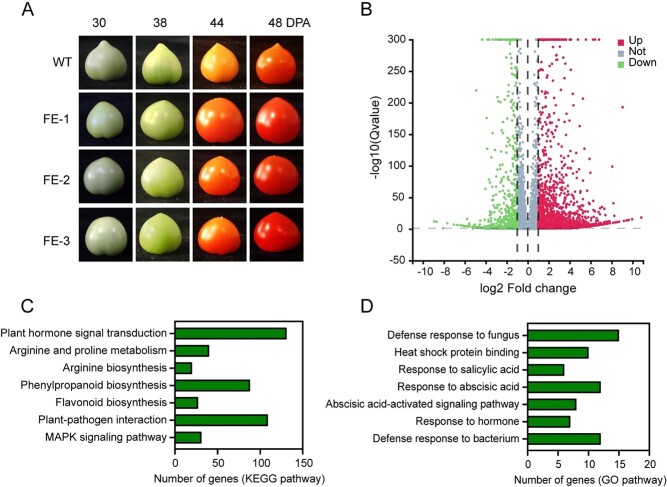
The creation of *SlMYB1*-FE lines and gene expression profiles of WT and *SlMYB1* FE lines. **A** Fruit color at different ripening stages in WT and T2 *SlMYB1* FE lines; DPA, days post-anthesis. **B** Volcano map of DEGs in WT and *SlMYB1*-FE lines. **C** KEGG annotation classification of DEGs in WT and *SlMYB1*-FE lines. **D** GO annotation classification of DEGs in WT and *SlMYB1*-FE lines.

*AtMYB1* is a type of R2R3-MYB transcription factor [31], which is suggested to protect *Arabidopsis* from in biotic and abiotic stress [32]. In this study, we identified the R2R3-MYB gene, *SlMYB1*, which is homologous to *AtMYB1* of tomatoes. We further proved that *SlMYB1* promoted lycopene synthesis by directly binding to the promoter of the lycopene synthesis-related genes, *SlLCY1* and *SlPSY2*. Meanwhile, the fruit-specific expression of *SlMYB1* could improve other nutritional quality traits, such as the contents of soluble sugars, soluble solids, and flavonoids, suggesting its multiple functions in the regulation of fruit nutritional quality. *SlMYB1* could also bind to the promoter of the pathogen-related gene *SlPR5* and thus improve the resistance to *B. cinerea.* Additionally, the fruit-specific expression of *SlMYB1* promoted the diversity of fruit types to maintain the physical integrity of fruit during transportation. Taken together, our findings revealed that the multi-functional MYB transcription factor *SlMYB1* regulated both lycopene synthesis and resistance in tomato plants by binding to the promoters of key regulators of different pathways. There are fewer studies on tomatoes, providing a serious theoretical basis for accelerating the excavation of genes involved in the control of tomato fruit quality.

## Results

### Features of MYB transcription factor *SlMYB1*

To explore the structural characteristics of the *SlMYB1* gene, we used the multiple sequence alignment of *SlMYB1* and its orthologs in different plant species, which showed that *SlMYB1* has a conserved R2R3 domain and thus, it is a typical R2R3-MYB protein (Fig. 1A). In addition, the phylogenetic analysis in tomato and *Arabidopsis thaliana* indicated that in MYB gene family *SlMYB1* and *SlMYB109* has the highest homology with *AtMYB1* (Fig. S1, see online supplementary material).

Real-time quantitative PCR (RT-qPCR) was then used to analyse the expression patterns of the *SlMYB1* and *SlMYB109* genes in different tissues of tomato plants, including roots (Rt), stems (St), leaves (L), and flowers (F), at different fruit developments stages, including mature green (MG), turning (T), orange (O), and red (R). The results indicated that *SlMYB1* expressed highly in roots and mature fruits, and also expressed in other analysed tissues (Fig. 1B). With increasing maturity, the expression level of *SlMYB1* also continuously increased (Fig. 1B), indicating that *SlMYB1* might have essential functions during the maturity stage. However, no significant difference was detected in the expression of *SlMYB109* in diverse tissues, and moreover the expression decreased slightly during fruit ripening (Fig. S2, see online supplementary material).

In order to identify the subcellular localization of *SlMYB1*, we constructed the recombinant plasmid *SlMYB1*-GFP, then expressed the plasmid transiently in tobacco leaf cells. As a result, the fluorescence of the *SlMYB1*-GFP fusion protein was detected in the nucleus, which was then co-localized with the DAPI staining (Fig. 1C), acknowledging the possibility that *SlMYB1* might be a transcription factor. To verify whether *SlMYB1* has transcriptional activity, the CDS (a coding sequence of amino acids in a protein) of *SlMYB1* was amplified and ligated to the GAL4 DNA-binding domain in the pGBKT7 vector. *SlMYB1* was transformed with the yeast expression vector pGBKT7, the negative control was pGBKT7 vector and the positive control was OsASR2-pGBKT7 which have been confirmed has transcriptional activity [33]. Results showed that the yeast transformed with *SlMYB1* could grow on the SD-Trp-His medium, suggesting that *SlMYB1* has transcriptional activity (Fig. 1D). Combined with previous bioinformatics results (Fig. 1A; Fig. S1, see online supplementary material), therefore, it might be a multi-functional transcription factor.

### Gene expression profiles of WT and *SlMYB1* FE lines

With a significant increase in the gene expression at fruit developmental stages, we used a fruit-specific promoter E8 [34] linked to the coding sequence (CDS) regions of *SlMYB1* to further explore the function of *SlMYB1* during fruit development. We generated the transgenic lines fruit-specific expression (FE) MYB1 in the fruit (Fig. 2A). The lines were further detected by RT-qPCR. FE lines expression levels were markedly regulated compared to wild-type (WT) (Fig. S3, see online supplementary material).

To systematically analyse the effects of the specific expression of *SlMYB1* in fruit stage of tomato, transcriptome analysis was performed to detect gene expression in WT and FE lines at the red ripening stage. First, we used RT-qPCR to verify the transcriptome results (Fig. S4, see online supplementary material). A total of 9864 genes expressed in different levels, 5083 genes were up-regulated, and 4781 genes were regulated in a down level (Fig. 2B) in FE lines. DEGs were subjected to KEGG and GO analyses for functional annotation. Phenylpropanoid biosynthesis, arginine biosynthesis, flavonoid biosynthesis, and other secondary metabolic pathways were enriched significantly. Plant–pathogen interaction pathways, such as the MAPK signaling pathway, and those related to defense responses to fungi and other pathogens were also enriched significantly (Fig. 2C and D). Furthermore, phenylpropanoid metabolism-related genes and defense responses genes in tomatoes were selected to visualize expression levels, which were found to be significantly up-regulated in *SlMYB1* FE lines (Fig. S5, see online supplementary material). Consequently, the transcriptome analysis indicated that *SlMYB1* might participate in both the biosynthesis of secondary metabolism and disease resistance.

### *SlMYB1* positively regulated the accumulation of lycopene

**Figure 3:**
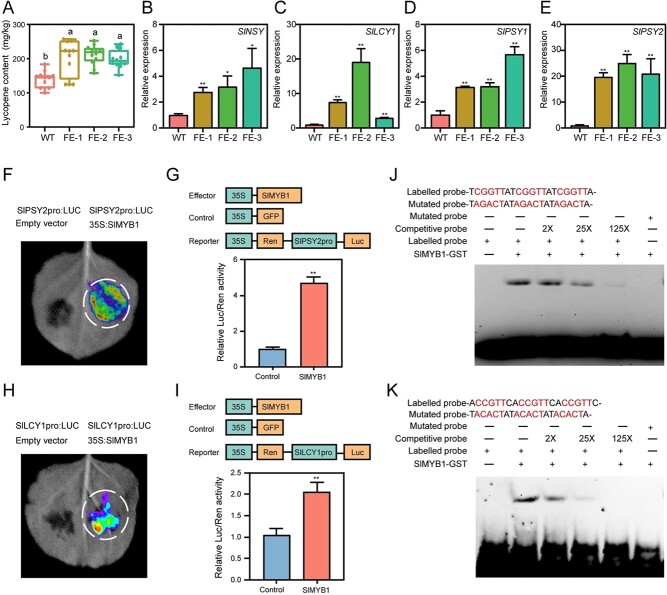
Mechanism analysis of *SlMYB1* regulating lycopene content. **A** Determination of the lycopene content in WT and T1 *SlMYB1* FE lines; letters indicate statistical significance by the Tukey’s test using GraphPad Prism (*n* = 11). **B**, **C**, **D**, and **E** Relative expression of lycopene biosynthesis-related genes *SlLCY1*, *SlNSY, SlPSY1*, and *SlPSY2* in WT and T2 *SlMYB1* FE lines; the error bars indicate stand for the SD (*n* = 3). Statistical significance was determined using the two-sample Student’s *t*-test (^*^P < 0.05 and ^**^P < 0.01). **F** and **H** Transient expression analysis of transcription factors showing that *SlPSY2* and *SlLCY1* were activated by *SlMYB1* in epidermal cells of *N. benthamiana* leaves; **G** and **I** Measurement of the luciferase activity using a luminometer; the error bars indicate stand for the SD (*n* = 3). Significance difference was determined using the Student’s *t*-test (two-samples, ^*^P < 0.05 and ^**^P < 0.01). **J** and **K** EMSA was carried out to validate the ability of the recombinant protein *SlMYB1*-GST to *in vitro* bind to the *cis*-element of the promoters of *SlPSY2* and *SlLCY1* genes. The negative controls were mutant probes.

Considering that the fruit colors of FE lines were significantly redder than WT lines (Fig. 2A), we wonder if this difference was due to the increase in lycopene. Results showed that the lycopene content in FE lines was significantly higher. Compared to the wild type, fruit lycopene content of the FE lines showed in a higher level, increased by 1.36–1.51 times (Fig. 3A).

Consistent with previous research carried out by Adaskaveg *et al.* [35] and Zhu *et al.* [36], in this study, several lycopene synthesis-related genes such as *SlLCY1*, *SlNSY*, *SlPSY1*, and *SlPSY2* [35, 36] were significantly up-regulated in RNA-seq (Fig. S5, see online supplementary material), which were further confirmed by RT-qPCR (Fig. 3B–E).

A special *SlMYB1* binding motif of CWGTT (W = C/G) was predicted by the PlantTFDB database (http://planttfdb.gao-lab.org/), and the analysis of the promoters of lycopene synthesis-related genes indicated the presence of this conserved motif located in all promoters of these genes (Fig. S6, see online supplementary material). To verify whether *SlMYB1* could activate target genes, the promoter of the lycopene synthesis-related gene was inserted into the pGreen0800-LUC vector to construct *SlPSY2*pro: LUC and *SlLCY1*pro: LUC, and the CDS region of MYB1 was inserted into pCXUN-HA to construct the 35S: *SlMYB1* vector. The vector was then transferred to the *GV3101* and infiltrated tobacco leaves. The luciferase assay revealed that *SlMYB1* remained and activated the target genes *SlPSY2* and *SlLCY1*, showing a higher LUC/REN ratio than control (Fig. 3F–I). The MYB1 binding motif was then labeled with biotin, and the CDS region of MYB1 was inserted into the pGEX-4 T-1 vector. The EMSA (Electrophoretic Mobility Shift Assay) experiment was carried out by obtaining the MYB1-GST recombinant protein by a prokaryotic expression system. Similar results reported that *SlMYB1* was directly bound to the *cis*-acting element in *SlLCY1* and *SlPSY2* (Fig. 3G and K). In short, *SlMYB1* was directly bound to the promoters of *SlLCY1* and *SlPSY2* and promotes these genes’ transcriptional expression to regulate lycopene accumulation.

### The *SlMYB1*-FE lines increased the contents of flavonoids, carotenoids, soluble sugars, and soluble proteins in tomato

**Figure 4:**
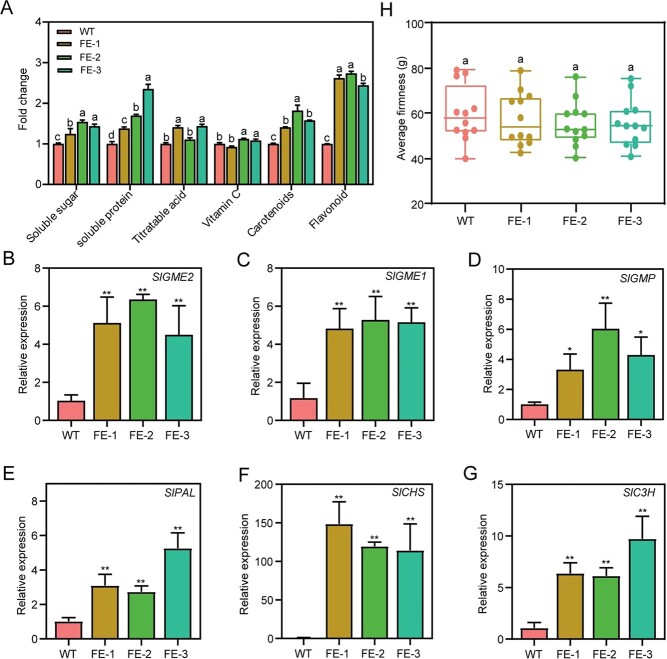
Analysis of partial fruit quality and related genes regulated by *SlMYB1* in tomato. **A** Determination of the contents of substances related to fruit quality in WT and T2 *SlMYB1* FE lines; error bars show the SD (*n* = 3). Letters indicate statistical significance by Tukey’s test using the GraphPad Prism software. **B**, **C**, and **D** The relative expression of the Vitamin C biosynthesis-related genes *SlGME1, SlGME2, SlGMP* in WT and T2 *SlMYB1* FE lines. **E**, **F**, and **G** The expression level of the flavonoid biosynthesis genes *SlPAL, SlCHS, SlC3H* in WT and T2 *SlMYB1* FE lines; the error bars stand for the SD (*n* = 3). Significant difference was determined using the Student’s *t*-test (two-samples, ^*^P < 0.05 and ^**^P < 0.01). **H** Measurement of fruit hardness in WT and FE lines; letters indicate statistical significance by the Tukey’s test with the GraphPad Prism software (*n* = 10).

Fruit quality is one of the most important tomato characteristics, associated with transcriptional reprogramming of multiple metabolisms. Due to its main expression in the fruit and participation in multiple metabolic pathways (Fig. 2C and D), *SlMYB1* might be related to tomato fruit nutritional quality, which itself was closely linked to metabolic pathways. Except for lycopene, we then tested several quality-related parameters, including soluble sugar, soluble protein, titratable acid, vitamin C, carotenoids, and flavonoids in WT and FE lines during the red ripening stage. Then we compared the content of flavonoids with WT lines, the results showed that the FE lines had a high rate of increase and increased 2.62, 2.74, and 2.44 times, respectively. The content of carotenoids also increased significantly, by 1.41, 1.81, and 1.58 times, respectively. For vitamin C, only FE-2 and FE-3 were slightly up-regulated by 1.19 and 1.17 times. Titratable acid was similar: line 1 and line 3 increased by 1.41 times and 1.43 times. The results of soluble sugar showed that all the lines were improved, which increased by 1.24, 1.54, and 1.44 times, respectively. The same was true for soluble proteins, which were 1.38, 1.69, and 2.34 times than WT (Fig. 4A).

Meanwhile, we selected some genes involved in flavonoids synthesis (*SlPAL*, *SlCHS*, and *SlC3H*) [37] and vitamin C (*SlGME1, SlGME2,* and *SlGMP*) [38] and performed RT-qPCR (Fig. 4B–G); these genes showed an up-regulated expression level. Further, we compared the fruits hardness between WT and FE lines during the red ripening stage and found no significant difference (Fig. 4H).

### *SlMYB1* positively regulated resistance to *B. cinerea* of tomato fruit

**Figure 5:**
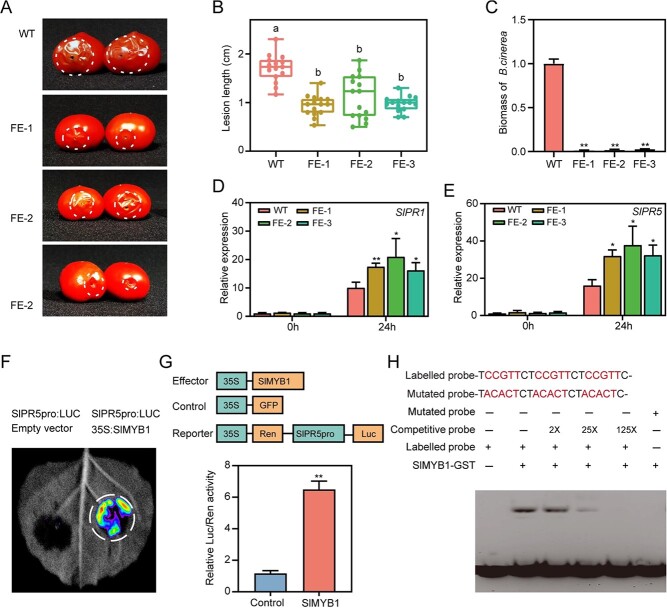
Mechanism analysis of *SlMYB1* regulating the resistance of *B. cinerea* in fruit. **A** Symptoms of *B. cinerea*-infected fruits of tomato WT and T2 *SlMYB1* FE lines 3 days later. **B** Statistics of the lesion length of fruit between WT and T1 *SlMYB1* FE lines of inoculation by *B. cinerea* after 3 days; letters indicate statistical significance by the Tukey’s test with the GraphPad Prism software (*n* = 15). **C** The *B. cinerea* biomass of WT and T2 *SlMYB1* FE lines after 3 days of inoculation with *B. cinerea*; values are means ±SD (*n* = 3). **D** and **E** Expression level of *SlPR1* and *SlPR5* after 24 h post-inoculation (hpi) by *B. cinerea* in WT and T2 *SlMYB1* FE lines; the gene expression levels were evaluated by RT-qPCR. **F** Transient expression analysis of transcription factors showing that *SlPR5* was activated by *SlMYB1* in *N. benthamiana* leaves epidermal cells. **G** Measurement of the luciferase activity by a luminometer; the error bars indicate stand for the SD (*n* = 3). Significance difference was determined using the Student’s *t*-test (two-samples, ^*^P < 0.05 and ^**^P < 0.01). **H** The recombinant protein *SlMYB1*-GST was able to *in vitro* bind to the *cis*-element of the *SlPR5* promoter and was validated by EMSA. The negative controls were mutant probes.

Tomato fruits are susceptible to being infected by fungi; *B. cinerea* is the most common fungal diseases during the maturation process and after harvesting [18]. Transcriptome analysis revealed that the *SlMYB1* FE lines were enriched in several disease resistance-related pathways (Fig. 2C and D), the expression level of several genes related in disease resistance was up-regulated (Fig. S5, see online supplementary material). It suggested that *SlMYB1* might be related to the disease resistance of tomato plants. Thus, *B. cinerea* inoculated the tomato plants at the red ripening stage, and we found that the lesion length in FE lines was reduced significantly. The mean lesion length of the wild type was 1.7 cm, and that of the FE lines was 0.94, 1.11, and 0.97 cm (Fig. 5A and B). Simultaneously, qPCR was used for the biomass quantification of *B. cinerea* in infected tomatoes, and significantly reduced biomass in FE lines was observed (Fig. 5C). Moreover, the expression level of the pathogen-related genes *SlPR1* and *SlPR5* were also increased in *SlMYB1* FE lines significantly after pathogen inoculation, compared to that in WT lines (Fig. 5D and E).

Further analysis of the *SlPR5* promoter revealed the presence of the *cis*-element bound by *SlMYB1*, leading to the notion that *SlMYB1* might activate the expression of *SlPR5* by binding to the promoter of *SlPR5*, which was linked to the pGreen0800-LUC vector (*SlPR5*pro: LUC), and tobacco leaves were then infiltrated with 35S: *SlMYB1*. *SlMYB1* was found to activate the expression of the reporter genes by the luciferase assay (Fig. 5F and G). Further, EMSA experiments confirmed that *SlMYB1* is directly bound to the CCGTT motif of the *SlPR5* promoter (Fig. 5H). The above results suggested that the fruit-specific expression of *SlMYB1* improved the resistance of tomato fruits to the fungi, *B. cinerea,* by binding the promoter of *SlPR5* directly, which was conducive to the healthy ripening of tomato fruits during the post-harvest storage.

### Fruit-specific expression of *SlMYB1* changed the shape of tomato fruit

**Figure 6:**
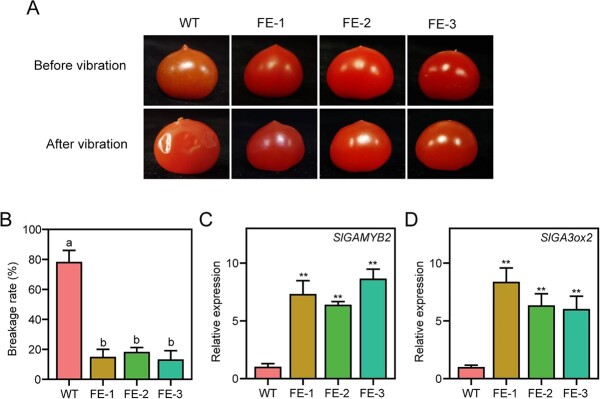
Effect of *SlMYB1* on fruit shape of tomato. **A** The mechanical treatment of WT and T2 *SlMYB1* FE lines. **B** Statistics of the breakage rates of WT and T2 *SlMYB1* FE lines; the error bars indicate stand for the SD (*n* = 10). Letters indicate statistical significance by Tukey’s test using the GraphPad Prism software. **C** and **D** Expression of *SGAMYB2* and *SlGA3ox2* genes in WT and T2 *SlMYB1* FE lines; the error bars indicate stand for the SD (*n* = 3). Significant difference was determined using the Student’s *t*-test (two-samples, ^*^P < 0.05 and ^**^P < 0.01).

Fruit shape is an important indicator of fruit quality. When evaluating the fruit characteristics, we found that the shape of the tomato fruit in FE lines had changed dramatically. After the fruit-specific expression of *SlMYB1*, the tomato fruit top shape was smooth or flat, rather than with sharp protruding edges (Figs 2A and 6A). The collision and friction during transportation often caused tomato fruit damage, resulting in the decay of tomatoes. We then conducted the random vibration experiments in accordance with international testing standards for transportation to prove whether the fruit top shape after the fruit-specific expression of *SlMYB1* is associated with fruit skin damage caused by vibration and fruits with smooth top shape have slighter skin damage. Analysis of breakage rate showed that the FE lines of *SlMYB1* indeed significantly reduce the risk of skin damage (Fig. 6A and B), which resulted in improved tomato fruit quality during transportation and storage.

**Figure 7:**
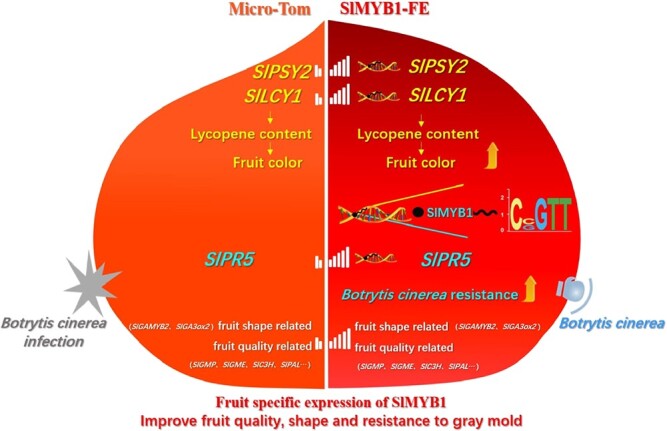
The use of *SlMYB1* as a working model for improving tomato fruit quality and resistance to gray mold. During ripening, the expression of *SlMYB1* increased continuously, which resulted in promoting downstream target genes expression level, *SlLCY1*, *SlPSY2*, and *SlPR5*, increasing the content of lycopene, thus making tomato fruits turn red, increasing the contents of flavonoids and other secondary metabolites, and consequently improving tomato fruit quality and resistance to *B. cinerea* and changing the fruit shape, making it flatter.

We found up-regulated expression of *SlGAMYB2* and *SlGA3ox2* in DEGs in the transcriptome. A recent study found that the overexpression of the gibberellin (GA)-related genes *SlGAMYB2* and *SlGA3ox2* could positively regulate the content of GA and produce fruits of similar shape [39]. We then quantitatively detected the expression of *SlGAMYB2* and *SlGA3ox2*s in *SlMYB1* FE lines, which were found to be up-regulated to a significantly higher level (Fig. 6C and D). However, luciferase assays showed that *SlMYB1* did not directly regulate the activation of *SlGAMYB2* and *SlGA3ox2*s (Fig. S7, see online supplementary material), suggesting that *SlMYB1* might regulate the fruit shape by regulating *SlGAMYB2* and *SlGA3ox2* indirectly.

## Discussion

Tomatoes are considered both fruits and vegetables, containing a variety of nutrients and thus greatly contribute to humans’ daily intake. However, tomato plants face a variety of biotic and abiotic stresses in different stages such as growth, development, and post-harvest storage [40], which consequently affect their yield and quality. After maturity, they are also affected by rot pathogen infection, leading to massive economic losses [41]. Here, we identified *SlMYB1* as a transcription factor with a wide range of functions in tomato, which plays an important role in improving both nutritional quality and resistance to *B. cinerea* in tomato fruit (Fig. 7).

MYB protein family as a huge transcription factor family contains many members that regulate plant functions such as development and defense responses. MYB family is also associated with a variety of biological pathways involved in both primary metabolism and secondary metabolism [42–44]. The fruit ripening process is also a transcription reprogram [45]. The expression levels of numerous genes were changed and concentrations of corresponding metabolites, the color and flavor of tomato fruits are continuously improved [46–48]. The brighter colors, better flavor, and quality of tomato plants can improve palatability and thus attract animals to feed. Thus, promoting the spread of seeds away from the plant and the production of many secondary metabolites can regulate stresses and develop signal responses in the plant. The fine flavor and quality of tomato fruits also attract humans, and secondary metabolites such as flavonoids and carotenoids have potential health benefits, including reduced risk of disease [49, 50]. Previous studies have shown that *SlMYB72* as a tomato MYB transcription factor regulates chlorophylls, flavonoids and carotenoids in fruits by directly targeting the related genes [27]. Our results proved that *SlMYB1* bind to the *SlLCY1* and *SlPSY2* promoters directly, and also increase the accumulation of lycopene at the red ripening stage.

MYB-like transcription factors may have some redundant functions in regulating the metabolism of lycopene; therefore, a greater number of them may be involved in this process. The mechanism by which developmental stability is achieved is called canalization [51]. The contents of many other metabolites were elevated in *SlMYB1*-FE lines including soluble sugars, soluble proteins, flavonoids and the expression of the associated synthetic genes also increased. Whether *SlMYB1* regulates the expression level of these substance synthesis genes needs further verification. We speculate that *SlMYB1* may act as other roles involved in other pathways to regulate the synthesis of these substances, indirectly affecting their content, or it may be because *SlMYB1* increases the content of polyphenols such as lycopene, enhances the antioxidant activity, and indirectly increases the content of these substances. It is speculated that *SlMYB1* is probably a component with a great ability to regulate nutrient levels and has great potential for future research, but further research is required to investigate the regulatory mechanisms of other metabolites. We also speculate that *SlMYB1* has various functions, including the regulation, synthesis, and also utilization of other metabolites, but further experiments are needed.

The fruit-specific expression of *SlMYB1* improved resistance to gray mold in tomatoes at the red ripening stage, which is believed to be mainly due to two reasons: firstly, by directly targeting *SlPR5*, and secondly, by regulating the expression of *SlPR1* that is indirectly involved in SAR and the regulation of phytohormones. We found that *SlMYB1* could bind the promoter of the *SlPR5* gene directly and increase its expression, which resulted in improved resistance to *B. cinerea. SlPR5* is a Thaumatin-like protein (TLP) and one of the key genes acting in plant resistance. The expression of TLP genes can be induced by a variety of stresses. Studies have reported that TLP proteins have antifungal activities, and transgenic plants expressing *TLP* genes have shown significantly increased tolerance and resistance to fungal pathogens. However, the molecular mechanism of antifungal activity of the TLP protein and its specific biological functions are not well understood [52]. Thus, our findings provide essential evidence suggesting that the MYB transcription factor might be an essential upstream regulator associated with TLP-encoding genes that confer dominant resistance to plant diseases. In addition, the transcript level of *SlPR1* changed in *SlMYB1* FE lines after treatment with *B. cinerea*. However, the mechanism of *SlPR1* remains elusive. The existing results showed that *SlPR1* is a leading participant in SAR [53]. Therefore, *SlMYB1* may play a vital role in the establishment of SAR against *B. cinerea.* Besides, transcriptome analysis showed the differential expression level in phytohormones pathway, including JA and SA, demonstrating that *SlMYB1* may participate in hormone signal transduction. It has been shown that *BjMYB1* enhanced *B. cinerea* resistance through activating the expression of chitinase, which bound to W-box-like elements [54]. *SlMYB1* also increased the *B. cinerea* resistance by regulating various signaling pathways in tomato plants.

Fruit shape is an important agronomic trait in tomato breeding. For vegetables and fresh fruits that are considered edible parts of plants, fruit shape is one of the most important external characteristics [55, 56]. Therefore, understanding the genetic regulation of fruit shape is of great importance for both basic and applied research. Our results indicated that the fruit-specific expression of *SlMYB1* could cause changes in fruit shape, making it smoother and flatter. We found up-regulated expression of *SlGAMYB2* and *SlGA3ox2* in DEGs in the transcriptome and a recent study reported that the MYB-like transcription factor *SlGAMYB2* also regulated tomato fruit shape with its target gene *SlGA3ox2* and caused similar changes in fruit characteristics during fruit development, resulting in the production of larger and flatter fruit than in WT plants [39]. Similar to the process of lycopene regulation, there may be more MYB proteins with conserved domains that regulate the fruit shape to cope with genetic mutations induced by environmental factors. Interestingly, the expression levels of *SlGAMYB2* and *SlGA3ox2* significantly increased in *SlMYB1* fruit-specific expression lines; however, further study revealed that *SlMYB1* cannot directly active these genes, indicating that it might have an indirect relationship with *SlGAMYB2* or its downstream target genes to regulate fruit shape.

In addition, *SlMYB1* was also strongly expressed in roots, suggesting that it may serve functions in roots. Transcriptome analysis revealed the different accumulation of a large number of secondary metabolites like spermidine (Fig. 2C and D), which was important in improving drought resistance [57], indicating that *SlMYB1* might also participate in drought stresses, which is required to be further confirmed by future experiments. What is more, it is well known that plant phenotypes are regulated by complex networks, as are fruit quality, fruit shape, and resistance. On the one hand, there must be a variety of conservative elements in the promoter of *SlLCY1, SlPSAY2, SlPR5*, so as to achieve fine regulation of expression; on the other hand, the binding sites of *SlMYB1* are also diversified, so as to achieve a wide range of regulation and can regulate multiple genes, and can avoid losing function due to the mutation of binding sites. We further predicted the other binding sites of *SlMYB1* by PlantTF DB and Plant Cistrome Database and confirmed the binding by EMSA (Fig. S8, see online supplementary material). The diversity of binding sites may be one of the reasons for the diversity of *SlMYB1* functions, but more evidence is needed to prove it.

In summary, *SlMYB1* was a transcription factor associated with tomato fruit quality and resistance to *B. cinerea*, and its expression gradually increased during the fruit ripening stage of tomato. The fruit-specific expression of *SlMYB1* resulted in improving the tomato fruit quality and resistance to *B. cinerea*, production of flatter fruits by changing the fruit shape, and enhanced part of the fruit nutritional quality of tomato plants during transportation and storage. In this study, we mainly focused on a potential candidate gene that will be helpful in breeding tomato varieties with high disease resistance and high quality in fruit by changing the expression level of only one gene.

## Materials and methods

### Plant materials and treatments

The cultivar ‘Micro-Tom’ was obtained and stored in a laboratory. Tomato plants were cultured in plastic pots with a diameter of 7 cm. The potting substrates were healthy soil and vermiculite (2:1). Plants were grown in a greenhouse with a standard as day/night cycle of 16 h/8 h and 25°C/20°C.

*B. cinerea* was cultured on the PDA medium incubated at 25°C in a week with a 14 h/10 h light/dark cycle [58]. The spores produced on the surface of the culture medium were collected by gently scraping with a brush and suspended in sterile water, and thereafter prepared spore suspension (2 × 10^5^ conidia mL^−1^). Using WT and *SlMYB1* fruit-specific expression tomato lines as materials, spore suspension was injected into the four sides of the fruit; each inoculum site received 10 μL of spore suspension. After being inoculated with the concentration, the plants were placed in a greenhouse for 24 h at 25 ± 2°C with 95% relative humidity [59].

In addition, the WT plants not inoculated with *B. cinerea* were used as control, and collected their various tissues, containing roots, leaves, flowers, stems, also fruits, at different maturity stages. The above samples were then frozen in liquid nitrogen and stored in an ultra-low temperature freezer at −70°C.

### RT-qPCR

The RT-qPCR experiment involved three biological replications. For each replicate, 0.1 g sample per plant was collected, and Plant RNA Kit R6827 (OMEGA) was used to extract the total RNA then reverse transcribed into cDNA with the UltraSYBR Mixture (CWBIO). RT-qPCR was performed by Applied Biosystems™ QuantStudio 6 Flex real-time PCR detection system. The 2^−ΔΔ^Ct method was used to calculate the gene differential fold of expression level [60], and tomato actin gene was chosen as control.

The biomass of *B. cinerea* was quantified based on the qPCR analysis method. Isolated genomic DNA from WT and FE plants was with the CTAB DNA extraction method. The 18S rDNA was used for the detection of *B. cinerea*, and the tomato actin gene was used as a control [61].

The primer sequences used are listed in Table S1 (see online supplementary material).

### Assay of subcellular localization and transcriptional activity

The coding sequence (CDS) of the *SlMYB1* gene was cloned into the pBIN-eGFP vector, and the fusion gene expression vector was constructed with the cauliflower mosaic virus (CaMV) 35S RNA promoter region. The *SlMYB1*-GFP vector was transformed into tobacco leaves, which were then infiltrated with the *GV3101* strain. The green fluorescent protein (GFP) fluorescence was observed by laser scanning confocal microscopy.

For the detection of transcription factor activity, the coding sequence (CDS) of *SlMYB1* was ligated into the pGBKT7 vector with the GAL4 DNA-binding domain to construct the vector of pGBKT7-*SlMYB1*. The constructed pGBKT7-*SlMYB1* and pGBKT7 vector as negative control were co-transformed into the yeast cells (AH109). Yeast cultivation was carried out on the medium without Trp (SD-Trp) or Trp and His (SD-Trp/His). Transcription activity was estimated based on the growth rates of yeast [33].

Table S1 (see online supplementary material) shows the primers used in this research.

### Plasmid construction and plant transformation

To construct a fruit-specific expression vector with the fruit-specific promoter for *SlMYB1*, the coding sequence (CDS) of *SlMYB1* was ligated to pX6-E8-*SlMYB1* to construct a recombinant vector. The transformed lines were obtained via *GV3101* (*Agrobacterium tumefaciens* strain). The *A. tumefaciens*-mediated method was chosen for plant transformation [62]. The identification of positive control was carried out by PCR and RT-qPCR, and then the positive lines T_2_ were used for the experiment.

Table S1 (see online supplementary material) shows the primer sequences.

### Measurement of tomato fruit quality

Tomato fruit quality traits, including soluble sugar, soluble solids, titratable acid, lycopene, flavonoid, etc., were evaluated at Shandong Yihui Testing Technology Company.

### Damage rate measurement

The WT and FE lines fruits at the stage of red ripening were placed in the same box and then on a random vibration shaker at a constant temperature of 25°C. After shaking for 10 h, the damage rates of fruits were determined according to international standards.

ISO 8318–2000 Packaging-Complete were used to carry out Sinusoidal vibration tests with variable frequency.

Table S1 (see online supplementary material) shows the primer sequences.

### Expression and purification of the protein

The CDS of *SlMYB1* was cloned into the prokaryotic expression vector pGEX4T-1 to construct the pGEX-4 T-1-*SlMYB1* fusion plasmid. The constructed fusion plasmid was transformed into *Escherichia coli* BL21 (DE3) and used 1 mmol L^−1^ of isopropyl-β-D-thiogalactoside (IPTG) to induce it at 37°C for 3 h. The culture supernatants were then collected. The *SlMYB1*-GST fusion protein was selectively separated and purified with glutathione.

### Electrophoretic mobility shift assay (EMSA)

To establish whether *SlMYB1* binds to the promoters of *SlPSY2*, *SlLCY1*, and *SlPR5*, an oligonucleotide probe containing the *SlMYB1* binding motif predicted by PlantTFDB (http://planttfdb.gao-lab.org/) was synthesized and labeled with biotin. Unlabeled oligonucleotide probes were used as competitors. EMSA was executed with the EMSA/Gel-Shift kit (China Biyuntian Biotechnology, Shanghai, China) according to the instructions.

Table S1 (see online supplementary material) shows the probe sequences.

### Luciferase reporter assay

The CDS of *SlMYB1* was inserted as an effector into the plasmid pCXUN-HA. The sequences of *SlLCY1*, *SlPSY2*, and *SlPR5* promoter were recombined into pGreenII 0800-LUC plasmid as reporter genes. The vector pBIN-EGFP was used as the negative control. The recombinant plasmid by *Agrobacterium*-mediated genetic transformation was then co-transformed to tobacco leaves and the NightShade LB 985 In Vivo Plant Imaging System was used for detecting both luminescence and fluorescence.

The luminescence intensity was detected by a luminometer, and the Dual-Luciferase Reporter Assay Kit (Vazyme, Nanjing, China) was used to perform the dual-luciferase reporter gene assay [63].

Table S1 (see online supplementary material) shows the primer sequences.

### RNA-Seq analysis

The WT and *SlMYB1*- FE lines fruits were collected at the red ripening stage for RNA-seq analysis, which was carried out in BGI Shenzhen. The analysis of enriched KEGG pathway and differentially expressed genes were performed based on the manufacturer’s standard procedure provided by BGI (BGI-Shenzhen, China), and the final visualization was done by TBtools software [64].

### Multiple sequence alignment and developmental genetic analysis

The NCBI database (https://www.ncbi.nlm.nih.gov/database) was utilized to identify *SlMYB1* homologous genes and download the tomato, *Arabidopsis*, potato, tobacco, and rice protein sequences. Multiple sequence alignments were implemented by MEGA6 software by the MUSCLE algorithm. Visual analysis was carried out by GeneDOC software.

Neighbor-Joining (NJ) was used to construct the phylogenetic tree, which is a distance-based method [65].

### Statistical analysis

The Tukey’s multiple comparison test or Student’s *t*-test was used for pairwise comparisons among the group means using the GraphPad Prism 9 software (GraphPad Software, San Diego, CA, USA, www.graphpad.com). The differences in the means of treatments were thought significant at 0.05 (^*^P < 0.05) and 0.01 (^**^P < 0.01).

### Accession numbers

The RNA-Seq raw data in our study was submitted with the accession number PRJNA791533 into the SRA database.

In this study the sequence data could be searched in the GenBank/Phytozome. The accession numbers are *SlMYB1* (Solyc09 g011780), *SlLCY1* (Solyc04 g040190), *SlNSY* (Solyc06 g074240), *SlPSY1* (Solyc03 g031860), *SlPSY2* (Solyc02 g081330), *AtMYB1* (AT3G09230), *AtMYB109* (AT3G55730), *StMYB122* (LOC102590968), *NtMYB122* (LOC107799770), *OsMYB1* (LOC4324801), *SlPAL* (Solyc09 g007900), *SlHCT* (Solyc03 g117600), *SlC3H* (Solyc10 g078240), *SlCHS* (Solyc09 g091510), *SlC4H* (Solyc06 g150137), *SlHQT* (Solyc07 g005760), *SlPR1* (Solyc09 g007010), *SlPR5* (Solyc08 g080660), *SlGAMYB2* (Solyc06 g073640), and *SlGA3ox2* (Solyc03 g119910).

## Supplementary Material

Web_Material_uhac282

## Data Availability

All data generated and analysed during this study are included in this article (and supplementary file).
