# Supplementary material for: SlMYB1 regulates the accumulation of lycopene, fruit shape, and resistance to Botrytis cinerea in tomato
Source: Hortic Res. 2022 Dec 22;10(2):uhac282. doi: 10.1093/hr/uhac282 (PMC9930398; doi:10.1093/hr/uhac282)

A

SlGAMYB2pro:LUC
Empty vector

SlGAMYB2pro:LUC
35S:SlMYB1

B

SlGA3ox2pro:LUC
Empty vector

SlGA3ox2pro:LUC
35S:SlMYB1

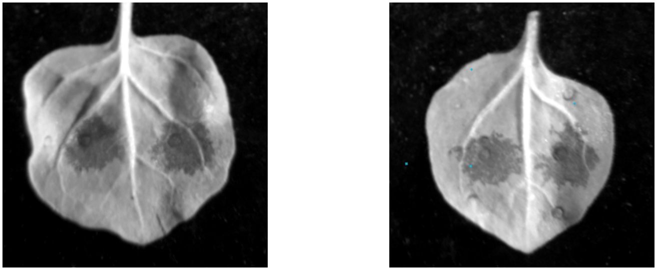

Supplement: Web_Material_uhac282 [file web_material_uhac282.zip › FigS7.pdf]
