# Supplementary material for: SlMYB1 regulates the accumulation of lycopene, fruit shape, and resistance to Botrytis cinerea in tomato
Source: Hortic Res. 2022 Dec 22;10(2):uhac282. doi: 10.1093/hr/uhac282 (PMC9930398; doi:10.1093/hr/uhac282)
Supplement: Web_Material_uhac282 [file web_material_uhac282.zip › FigS8.pdf]

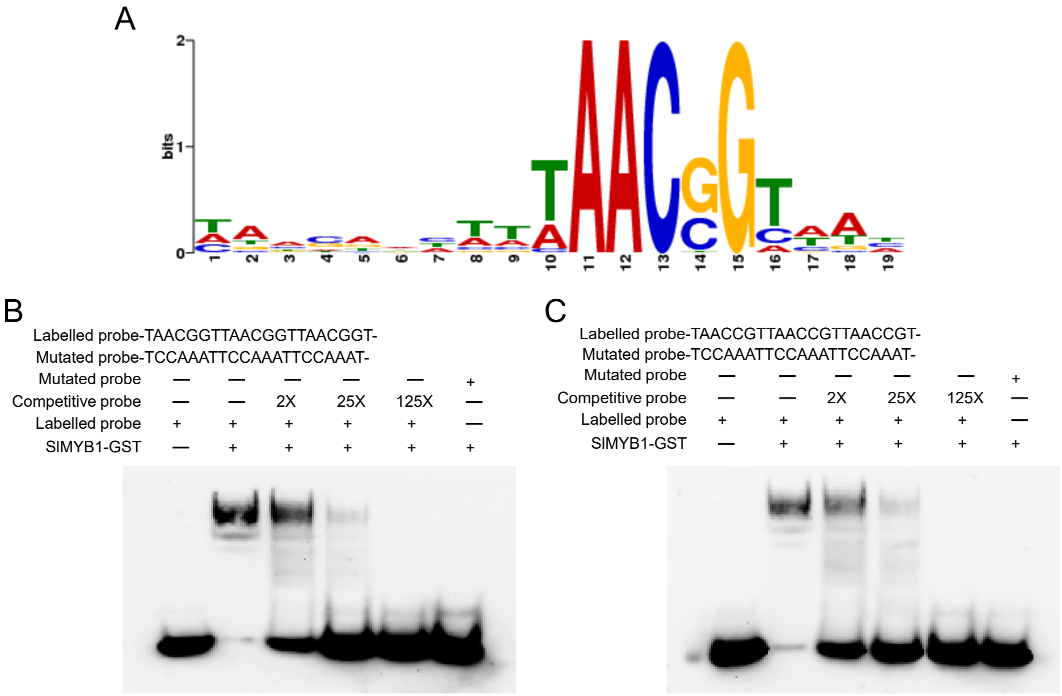
A
bits
B
Labelled probe-TAACGGTTAACGGTTAACGGT-
Mutated probe-TCCAAATTCCAAATTCCAAAT-
Mutated probe
Competitive probe
2X
25X
125X
Labelled probe
SlMYB1-GST
C
Labelled probe-TAACCGTTAACCGTTAACCGT-
Mutated probe-TCCAAATTCCAAATTCCAAAT-
Mutated probe
Competitive probe
2X
25X
125X
Labelled probe
SlMYB1-GST
