# Supplementary figures and images for: SlMYB1 regulates the accumulation of lycopene, fruit shape, and resistance to Botrytis cinerea in tomato
Source: Hortic Res. 2022 Dec 22;10(2):uhac282. doi: 10.1093/hr/uhac282 (PMC9930398; doi:10.1093/hr/uhac282)

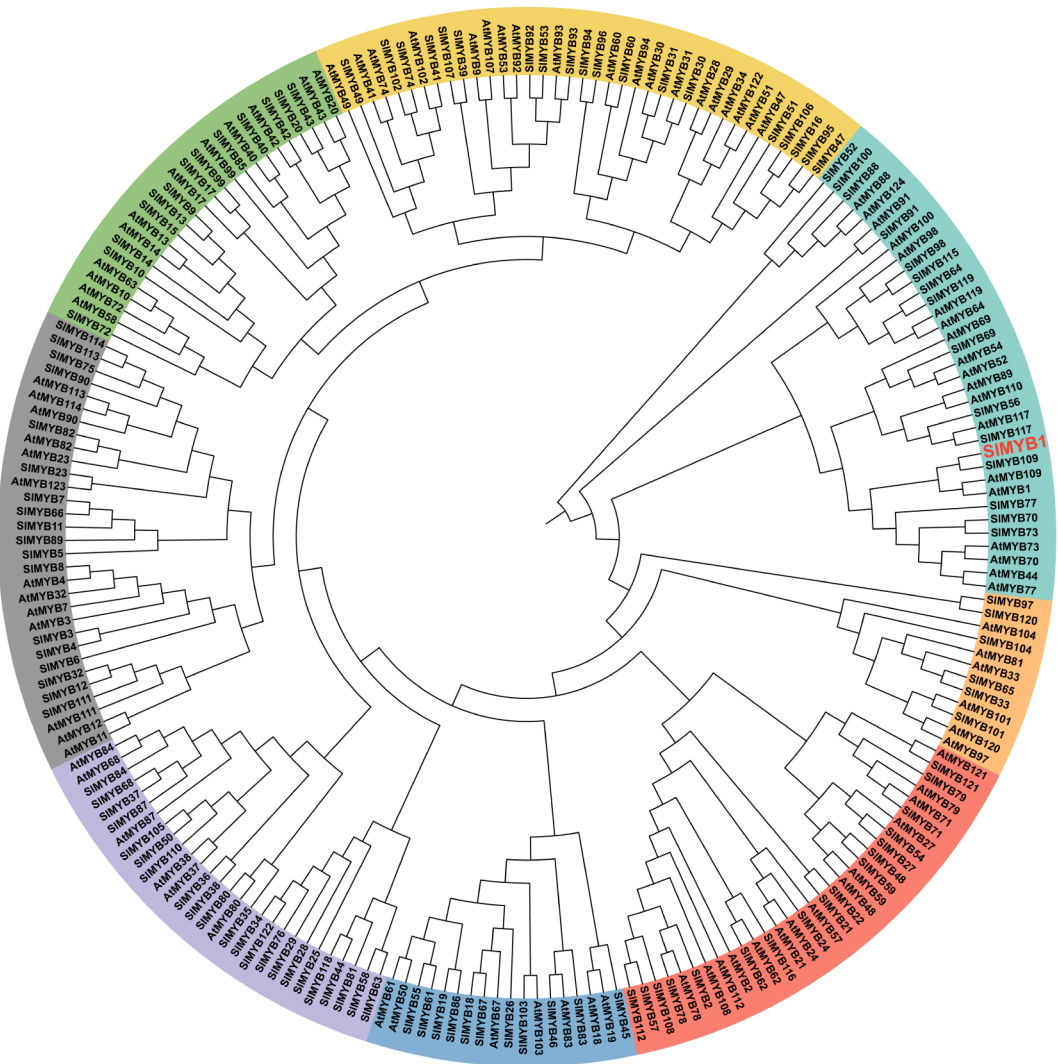

Supplement: Web_Material_uhac282 [file web_material_uhac282.zip › FigS1.pdf]

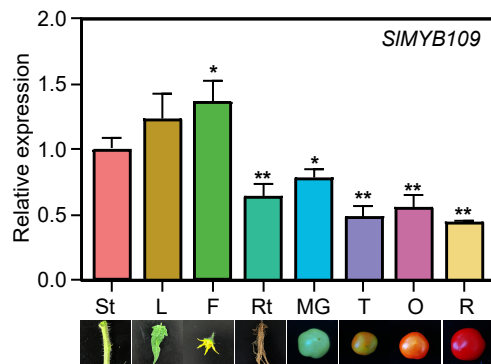

Supplement: Web_Material_uhac282 [file web_material_uhac282.zip › FigS2.pdf]

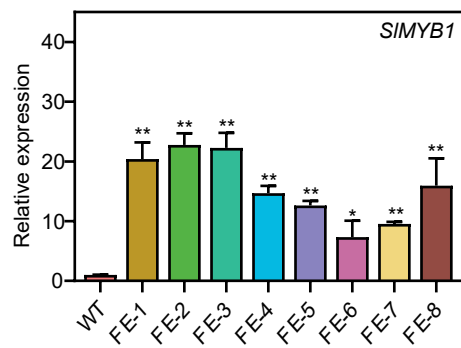

Supplement: Web_Material_uhac282 [file web_material_uhac282.zip › FigS3.pdf]

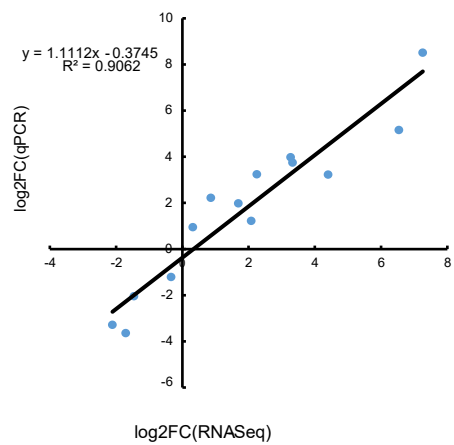

Supplement: Web_Material_uhac282 [file web_material_uhac282.zip › FigS4.pdf]

A

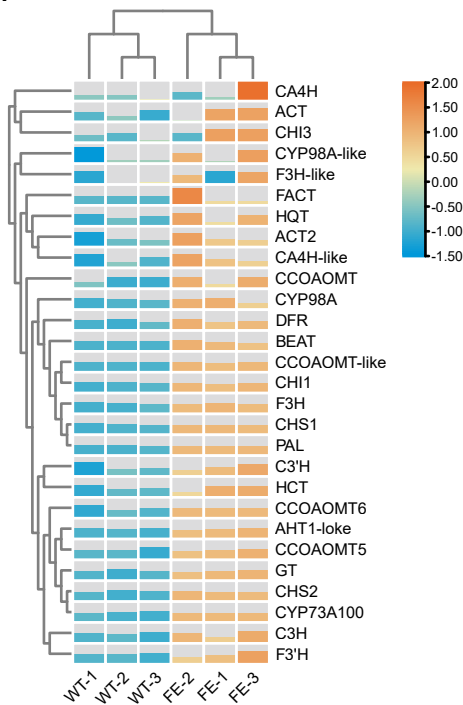

B

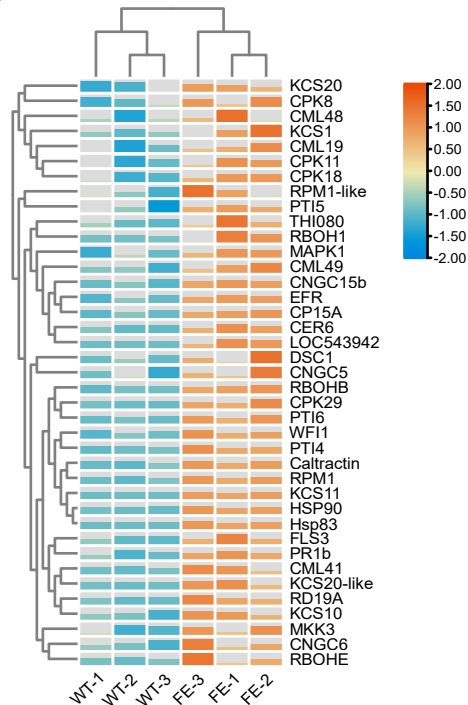

Supplement: Web_Material_uhac282 [file web_material_uhac282.zip › FigS5.pdf]

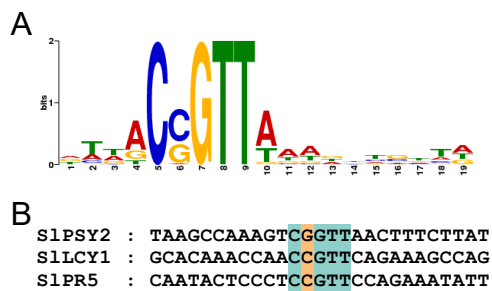

Supplement: Web_Material_uhac282 [file web_material_uhac282.zip › FigS6.pdf]
